# A synchrotron-based local computed tomography combined with data-constrained modelling approach for quantitative analysis of anthracite coal microstructure

**DOI:** 10.1107/S1600577514002793

**Published:** 2014-03-18

**Authors:** Wen Hao Chen, Sam Y. S. Yang, Ti Qiao Xiao, Sherry C. Mayo, Yu Dan Wang, Hai Peng Wang

**Affiliations:** a Shanghai Synchrotron Radiation Facility, Shanghai Institute of Applied Physics, Chinese Academy of Sciences, Shanghai 201204, People’s Republic of China; b Graduate University of Chinese Academy of Sciences, Beijing 100049, People’s Republic of China; c CSIRO Materials Science and Engineering, Private Bag 33, Clayton South, Victoria 3169, Australia; d Institute of Theoretical Physics and Department of Physics, Shanxi University, Taiyuan, Shanxi 030006, People’s Republic of China

**Keywords:** quantitative local CT, data-constrained modelling, coal, multi-spectrum X-ray CT

## Abstract

Quantifying three-dimensional spatial distributions of pores and material compositions in samples is a key materials characterization challenge, particularly in samples where compositions are distributed across a range of length scales, and where such compositions have similar X-ray absorption properties, such as in coal. Consequently, obtaining detailed information within sub-regions of a multi-length-scale sample by conventional approaches may not provide the resolution and level of detail one might desire. Herein, an approach for quantitative high-definition determination of material compositions from X-ray local computed tomography combined with a data-constrained modelling method is proposed. The approach is capable of dramatically improving the spatial resolution and enabling finer details within a region of interest of a sample larger than the field of view to be revealed than by using conventional techniques. A coal sample containing distributions of porosity and several mineral compositions is employed to demonstrate the approach. The optimal experimental parameters are pre-analyzed. The quantitative results demonstrated that the approach can reveal significantly finer details of compositional distributions in the sample region of interest. The elevated spatial resolution is crucial for coal-bed methane reservoir evaluation and understanding the transformation of the minerals during coal processing. The method is generic and can be applied for three-dimensional compositional characterization of other materials.

## Abstract 2

A quantitative local computed tomography combined with data-constrained modelling has been developed. The method could improve distinctly the spatial resolution and the composition resolution in a sample larger than the field of view, for quantitative characterization of three-dimensional distributions of material compositions and void.

## 1. Introduction   

Three-dimensional imaging of material compositions remains one of the most challenging aspects of materials characterization, and doing so quantitatively adds another level of complexity to this challenge. Quantitative computed tomography (CT) is emerging as a viable solution to this challenge; however, overcoming technical limitations has proven difficult, especially the ability to discriminate between three-dimensional distributions of minerals with similar X-ray absorption properties. Quantitative identification of fine features is also very difficult when their sizes are smaller than the X-ray CT resolution, such that a voxel (volumetric pixel, the minimum unit of the CT slice image) in the reconstructed CT image contains multiple compositions. The reconstructed CT value of the voxel represents a composition-weighted average. This is the so-called partial-volume effect (Ketcham & Carlson, 2001 ▶). Increasing X-ray CT resolution can reduce this effect, but the sample size will have to be decreased accordingly, which may either alter its fine structure or may not be possible at all owing to its fragility.

Another significant challenge encountered in X-ray CT is overcoming the limited field of view (FOV), which is dictated by hardware limitations, most notable from the size of the imaging detector. The consequence is that samples must either be physically trimmed to fit within the FOV, or the imaging resolution must be lowered to accommodate the sample, both of which are undesirable. A new imaging methodology, called local CT, has recently emerged and can overcome the FOV limitation of the detector. It can enable high resolution in X-ray CT imaging of large samples and has been broadly investigated in recent years (Delaney & Bresler, 1995 ▶; Katsevich & Ramm, 1996 ▶; Faridani *et al.*, 1992 ▶; Kudo *et al.*, 2008 ▶; Kyrieleis *et al.*, 2011 ▶). However, most local-CT algorithms are qualitative algorithms or are difficult to apply in practice because of restrictions, such as requiring global projections at certain projection angles and knowledge of the exact sample boundary. It is well know that CT reconstruction from an interior data generally leads to artifacts in slice in the form of a global (*i.e.* feature-independent) elevation of the gray values towards the edge of the reconstructed region of interest (ROI) (Lewitt & Bates, 1978 ▶). This kind of artifacts is due to the loss of low-frequency information which can be suppressed by projection extension (Lewitt, 1979 ▶). Pseudo-global tomography (Chen *et al.*, 2014 ▶) uses an effective strategy to compensate the lost part of projections in the local CT, which can effectively reduce DC-shift and low-frequency artifacts caused by projection truncation. It can efficiently perform quantitative local reconstruction with a standard calibration and so we have applied this method to our local-CT analysis.

Previously, data-constrained modelling (DCM) approaches have been applied in conjunction with synchrotron-based X-ray CT to quantitatively characterize material compositions distributions in samples and hydrocarbon reservoir characterizations (Yang *et al.*, 2007 ▶, 2008 ▶, 2010*a* ▶, 2010*b* ▶, 2013 ▶; Mayo *et al.*, 2012 ▶; Trinchi *et al.*, 2012 ▶). The DCM software is available at http://www.ict.csiro.au/downloads.php?swid=24. This approach utilizes X-ray absorption information obtained from multi-energy CT data as constraints to effectively distinguish between materials that have similar X-ray absorption properties, and allows composition distributions at length scales smaller than the X-ray CT resolution to be modelled as partial volumes.

In this study, the DCM approach and pseudo-global tomography are combined to form a new characterization approach, which is applied to synchrotron-based multi-energy X-ray local-CT datasets to investigate the three-dimensional compositional distributions and physical structure of a coal sample. Coal was chosen as the model sample owing to its worldwide significance as an energy source, and quantification of its volume and spatial disposition of pores, fractures and minerals, as well as visualizing that the physical structure of coal is a fundamental requirement for coal-bed methane reservoir evaluation and understanding the transformation of the minerals during coal processing. The high quality and monochromaticity of synchrotron-based X-ray light is optimal for quantitative CT reconstruction with a high resolution. The high brightness of synchrotron X-rays effectively reduces the sample exposure time, which can significantly reduce the impact of the experimental instabilities associated with time. In order to evaluate the advantage gained from local-CT-based DCM, global-CT-based DCM has also been performed. With the requirement of fitting the whole sample into the FOV of the detector, the CT resolution is low in global CT. The result shows that local CT can visualize more details of the sample, which provide more spatially resolved constraints for DCM than with global CT. It enables an accurate analysis of the three-dimensional distribution of coal compositions. Consequently, the combination of local CT and DCM can enable the partial volume effect to be characterized at a high spatial resolution.

## 2. Sample description and experimental parameters   

Coal is a complex polymeric material; its main physical compositions include: coal matrix (the organic compositions), void (pores and fractures) and mineral (inorganic constituents) (Levine *et al.*, 1982 ▶). In order to characterize and visualize the distributions of these compositions, various techniques have been used (Lawrie *et al.*, 1997 ▶; Bruening & Cohen, 2005 ▶; Vassilev & Tascón, 2003 ▶; Creelman & Ward, 1996 ▶; Galbreath *et al.*, 1996 ▶; Saikia & Ninomiya, 2011 ▶; Karacan & Okandan, 2001 ▶; Yao *et al.*, 2009 ▶). Among all these techniques, X-ray micro-CT has a unique advantage as a sample non-destructive three-dimensional characterization method.

The coal sample investigated in this study was collected from an underground mine in Yangquan, originating from Qinshui basin, which is one of the most gas-rich coal basins in China. It was carefully sanded by hand into a cylindrical shape with a diameter of 4 mm and a length of 10 mm. Table 1 ▶ shows a list of composition minerals and their overall volume percentages (Wang *et al.*, 2013 ▶). The total porosity of the coal sample was estimated as 4.43%.

When X-rays pass through the sample, the X-ray attenuation by each composition depends on both its volume fraction and its X-ray absorption coefficient. Since the typical noise level of X-ray CT is 1%, the mineral compositions can be ignored if their volume fractions are small enough such that they contribute much less than 1% of X-ray attenuation by the sample. According to this criteria, pyrite, dolomite, plagioclase and manganese dioxide can be ignored, and the remaining compositions of the sample are grouped as follows (Wang *et al.*, 2013 ▶):

(*a*) Void (group A);

(*b*) Coal matrix (group B);

(*c*) Illite, quartz and kaolinite (group C);

(*d*) Chlorite and titania (group D).

**Figure 1:**
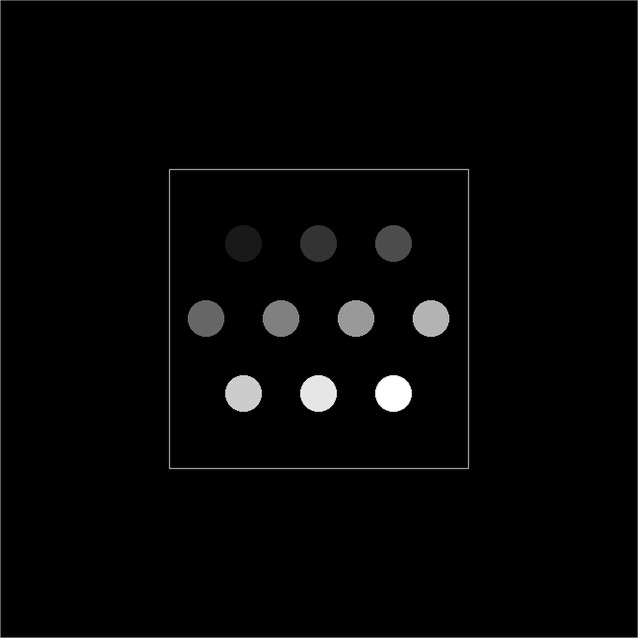
Schematic of the numerical phantom. The circular sub-regions are mixtures of coal matrix and one other main composition in various proportions (the volume fraction step is 10%). From left to right and top to bottom, the volume fractions of the other main composition are 10%, 20%,…, 90%, 100%. The background is the coal matrix. The square region indicates the region chosen to analyze the influence of different experimental conditions.

The accuracy of quantitative CT is related to X-ray CT experimental parameters, including CT resolution, the sample-to-detector distance (SDD) and X-ray beam energies. A pre-analysis is required to optimize the sample-specific experimental parameters. This pre-analysis is focused on the evaluation of impacts of SDD and X-ray beam energy in X-ray CT of a sample containing the compositions described above. The pre-evaluation was conducted with a numerical phantom with 680 × 680 × 1 voxels (a voxel has a size of 3.7 µm × 3.7 µm × 3.7 µm) (Fig. 1 ▶). As a parallel beam is used, one phantom slice is sufficient for the simulation. The numerical phantom is composed of circular sub-regions which comprise different proportions of coal matrix and one other main composition (void, group C minerals, or group D minerals) with volume fraction step of 10%. The background is coal matrix. The X-ray total linear absorption and phase-shift coefficients of main compositions (except void) at energies of 12 keV, 18 keV, 24 keV and 30 keV are listed in Table 2 ▶.

Projection images of the phantom were simulated with monochromatic parallel beams at X-ray energies of 12 keV, 18 keV, 24 keV and 30 keV. A series of SDDs were chosen in the simulation. Nine hundred projections over a total rotation angle of 180° around the *z*-axis (normal to the phantom slice) were obtained with a 0.2° angular step between projections at each X-ray energy. The projection images correspond mathematically to the Radon transforms of the phantom with X-ray absorption and refraction. The images contain phase-contrast effects for non-zero SDDs. That is, the projection images recorded by the detector were intensity images of the sample after Fresnel propagation at the SDD distance.

A typical noise level for X-ray CT of 1% Gaussian noise was added to the simulated X-ray projections. After the simulated projections were obtained, images were subjected to phase-retrieval processing prior to tomographic reconstruction. Phase-retrieval processing is required for the phase-contrast imaging mode used (non-zero SDD) and results in an apparent improvement in signal-to-noise level. Furthermore, it eliminates the phase-contrast ‘edge-enhancement’ effect to transform the data into a form suitable for quantitative analysis. Slices of X-ray total linear absorption coefficient at a given X-ray energy for the phantom sample were obtained by tomographic reconstruction.

The square region indicated in Fig. 1 ▶ was chosen to evaluate the performance of tomographic reconstruction under different experimental conditions. The normalized cross-correlation (NCC) between the reconstructed and original square region was calculated,
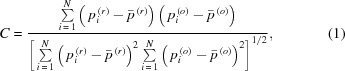
where 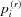 and 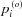 are the reconstructed and original total linear absorption coefficient values at the *i*th pixel, respectively, while 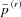 and 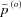 are their mean values. The NCC represents a template matching of two datasets, which means that a higher NCC value corresponds to a closer match between the predicted image and the real sample.

Table 3 ▶ gives the NCC values for different compositions and energies at a simulated noise level of 1% when SDD = 40 mm. It shows that different compositions are associated with different optimal energies. For a multi-composition sample, the optimal energy is a compromise between the optimal energies for the main compositions of the sample. For the coal sample in this paper, the optimal energy is near 18 keV. Considering that NCC is relatively insensitive to the beam energy and based on the experience about the X-ray stability on the BL13W beamline at Shanghai Synchrotron Radiation Facility (SSRF), the energies of 18 keV and 24 keV were chosen as the experimental energies.

**Figure 2:**
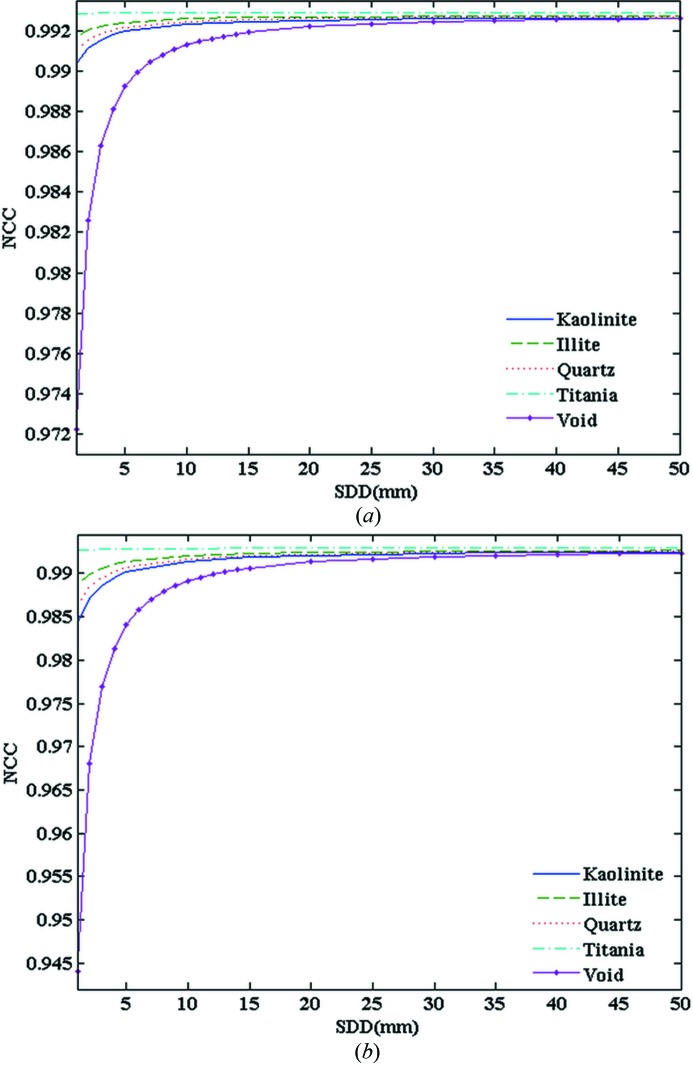
Relation between the NCC and SDD for different energies at experimental noise. (*a*) 18 keV energy; (*b*) 24 keV energy.

Fig. 2 ▶ shows that the NCC value first increases and then keeps constant with SDD. As SDD increases from zero, NCC increases. This is due to the fact that NCC is sensitive to noise, and phase-retrieval processing was applied to the non-zero SDD case which could improve the signal-to-noise of the CT reconstruction. The NCC almost keeps constant when SDD ≥ 30 mm. However, a larger SDD induces a higher level of Fresnel diffraction and edge enhancement effect which will affect the reconstructed absorption indices (Xiao *et al.*, 2005 ▶). In this paper, the SDD was chosen as 40 mm for experimental convenience with minimum deviation from the ideal case where NCC = 1.

## 3. X-ray CT imaging   

### 3.1. X-ray CT experiment   

The X-ray CT projection images were acquired on the BL13W beamline at SSRF. According to the previous analysis, monochromatic X-ray beam energies at 18 keV and 24 keV had been selected. The SDD was set at 40 mm.

BL13W is a wiggler beamline which produces monochromatic X-rays with narrow energy band (Δ*E*/*E* < 5 × 10^−3^, where *E* is the photon energy) *via* a Si(111) double-crystal monochromator. For the purpose of this study, it can be regarded as an ideal monochromatic beam.

For global CT, the combination of the CCD detector (with a native pixel size of 7.4 µm) and the optical lens (×2) provided an effective isotropic projection image with a pixel size of 3.7 µm. For each energy, 1080 global projection images were collected with equal angular spacing between each view with a total rotation angle of 180°. The local CT projection images were acquired under the same experimental conditions except with a ×10 optical lens, providing an effective pixel size of 0.74 µm. The available FOV places a limitation on the size of the sample region to be imaged in the local CT. The region was selected to be near the center region of the sample with a diameter of 1.49 mm. A total of 5400 local projection images were collected at each energy with a total sample rotation angle of 180° and equally spaced angular increment.

### 3.2. CT image reconstruction   

The projection images were pre-processed using *X-TRACT* software (Gureyev *et al.*, 2011 ▶). Paganin’s phase-retrieval algorithm (Paganin *et al.*, 2002 ▶) was applied with parameters tuned to minimize noise and edge enhancement without loss of resolution. A ring artifact correction was also applied to minimize the ring artifacts. For local CT, image slices of size 2013 × 2013 pixels were reconstructed by pseudo-global tomography (Chen *et al.*, 2014 ▶) from local projection images. Similarly, for global CT, image slices of size 2048 × 2048 pixels were reconstructed from global projection images.

**Figure 3:**
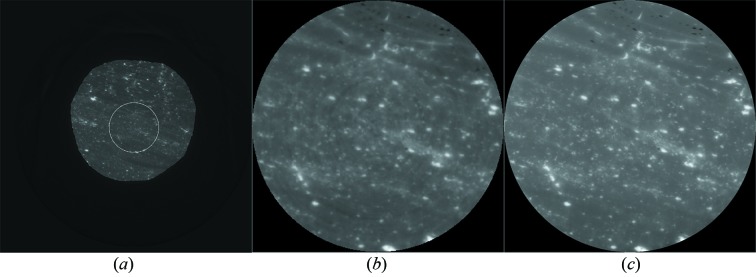
Typical two-dimensional CT reconstructed slices at X-ray energy 18 keV. (*a*) The low-resolution global reconstruction. The white circle indicates the ROI for the local CT. (*b*) Magnified view of the ROI in (*a*). (*c*) The high-resolution local reconstruction in the ROI on the same slice. The pixel intensity is proportional to the total linear absorption coefficient.

Fig. 3 ▶ shows typical two-dimensional CT reconstructed slices for global and local CT, in which there are some visible ring artifacts in the global CT. The gray areas in the reconstructed CT images (Fig. 3 ▶) are predominantly coal matrix, while the white areas are mainly minerals. By comparing Figs. 3(*b*) ▶ and 3(*c*) ▶ it is clear that, because of high resolution in the local CT, Fig. 3(*c*) ▶ provides more details in the ROI than Fig. 3(*b*) ▶. This gives fine spatially resolved constraints for DCM.

### 3.3. Constant shift correction   

**Figure 4:**
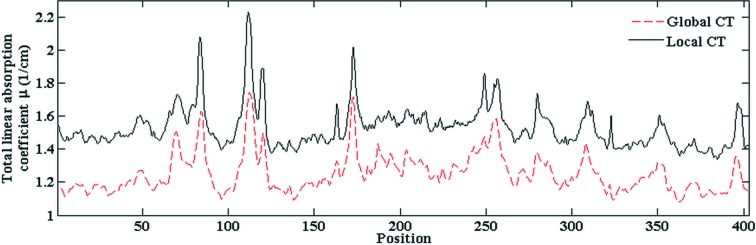
Central horizontal profiles of the ROI in Fig. 3 ▶ for local and global CT.

The reconstructed local CT images using pseudo-global tomography may differ by a constant shift in the pixel intensity, which is inevitable in local CT using the inverse Radon transform (Natterer, 1986 ▶). Fig. 4 ▶ shows the central horizontal profiles of the ROI shown in Fig. 3 ▶ for local and global CT. A constant offset is evident. In order to obtain the correct quantitative information, this constant shift must be corrected.

**Figure 5:**
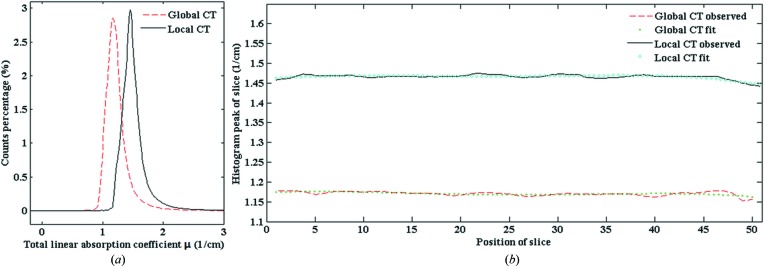
Typical histogram of one slice and the histogram peak as a function of slice position. (*a*) Typical histograms of one slice for global and local CT. (*b*) The histogram peak as a function of slice position for global and local CT and their fit curves.

**Figure 6:**
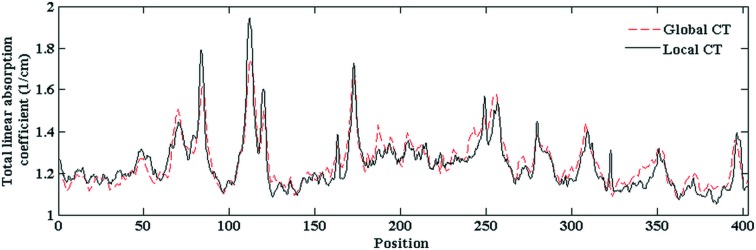
Central horizontal profiles of the ROI in Fig. 3 ▶ for local and global CT after constant shift correction.

This correction was performed as follows. Based on the histogram of the ROI in each slice, the absorption coefficient as a function of CT slice position was plotted and then fitted with a fourth-order polynomial (Fig. 5 ▶). By comparing the fitted curves for global and local CT, the shift between them was calculated. This calculated shift value was used to correct the offset. Fig. 6 ▶ shows the corrected result. The constant shift has disappeared.

## 4. Data-constrained microstructure prediction   

The reconstructed local CT images have a voxel size of 0.74 µm × 0.74 µm × 0.74 µm, while the reconstructed low-resolution global-CT images have a voxel size of 3.7 µm × 3.7 µm × 3.7 µm. Consequently, the features which were directly observable from CT images, such as pores, cannot be smaller than such a voxel size. The DCM approach can reveal fine features smaller than the CT image voxel size (Yang, 2012 ▶; Yang *et al.*, 2007 ▶, 2008 ▶, 2010*a* ▶,*b* ▶, 2012 ▶, 2013 ▶; Mayo *et al.*, 2012 ▶; Trinchi *et al.*, 2012 ▶) as compositional partial volumes in the same voxel by incorporating multiple sets of X-ray CT data acquired at different X-ray energies as constraints. Obviously, higher-resolution X-ray CT data provide more spatially resolved constraints for DCM, which would enable more accurate DCM characterization of microstructures. In other words, local CT would enable elevated DCM accuracy for regional microstructures. It overcomes the limitation of the FOV and enables high-resolution observation of the ROI in a large sample. In the DCM model a cubic grid of *N* = 2013 × 2013 × 245 voxels was used to represent the ROI in local CT, where 245 is the number of selected slices, while for global CT a cubic grid of *N* = 403 × 403 × 49 voxels was used to represent the same ROI, where 49 is the number of selected slices. The size of a voxel in the DCM model was equal to the voxel size of reconstructed CT images. For voxel *i* (*i* = 1, 2,…, *N*), where *N* is the total number of voxels in the system, the DCM nonlinear optimization approach was used to solve for the volume fraction values of compositions voxel by voxel. The approach is to minimize the following objective function (Yang, 2012 ▶),
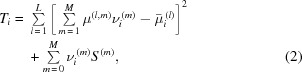
where *L* (= 2) is the number of X-ray CT data sets acquired from the sample at X-ray energies 18 and 24 keV; *M* (= 3) is the number of non-void compositions; 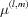 (*l* = 1, 2) are the expected X-ray total linear absorption coefficient values of composition group *m* at the two X-ray energies; 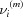 are volume fractions for composition group *m* on voxel *i*, and 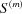 are the self-energy (phenomenological chemical potential) of composition group *m*; 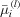 (*l* = 1, 2) are X-ray CT measured total linear absorption coefficient values on voxel *i* at the above X-ray energies. In equation (2), the first term represents the difference between the expected and CT-reconstructed X-ray total linear absorption coefficients, and the second term accounts for the chemical potential differences among different compositions. The interactions between compositions had been ignored considering the absence of further measurement data (Yang, 2012 ▶). In this paper, the chemical potentials were treated as adjustable parameters to reproduce the measured compositional volume fractions of the whole sample.

The minimization of equation (2) is solved using a constrained search algorithm (Yang, 2012 ▶) with the following constraints,
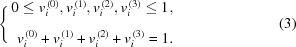
When the chemical potentials of groups A, B, C and D were set as 0.0002652, 4.5705 × 10^−5^, −0.001173 and −0.0103822, respectively, the computed overall volume fractions of compositions reproduced the measured value using helium gas porosimetry and the ash analysis process.

**Figure 7:**
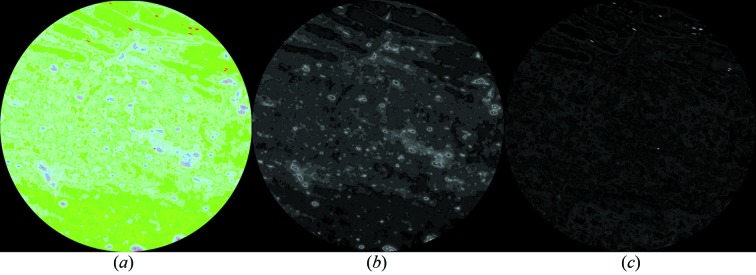
DCM results of the ROI in Fig. 3 ▶. In image (*a*), void is displayed as red, coal matrix is displayed as green, minerals of group C are displayed as blue, and minerals of group D are displayed as purple. Image (*b*) gives the distribution of group C, and image (*c*) gives the distribution of void. On a pixel, the displayed intensity for each colour is proportional to an appropriate compositional volume fraction. The co-existence of multiple compositions in a voxel is shown as colour mixing in (*a*).

Fig. 7 ▶ shows the DCM results on the same slice as in Fig. 3 ▶. The average compositional volume fractions are listed in Table 4 ▶. Obviously, there are some colour mixed regions in Fig. 7(*a*) ▶, indicating that multiple compositions coexist in these regions. For instance, the yellow (red mixed with green) pixels indicate the coexistence of void and coal matrix. Colour mixing also means that these voxels contain compositions smaller than the X-ray CT resolution. It should be noted that the colour mixing becomes less obvious when a voxel is dominated by one composition. Figs. 7(*b*) and Fig. 7(*c*) ▶ show a high occurrence of void near the boundary between the minerals group C and the coal matrix. This observation is consistent with the results of Wang *et al.* (2013 ▶).

**Figure 8:**
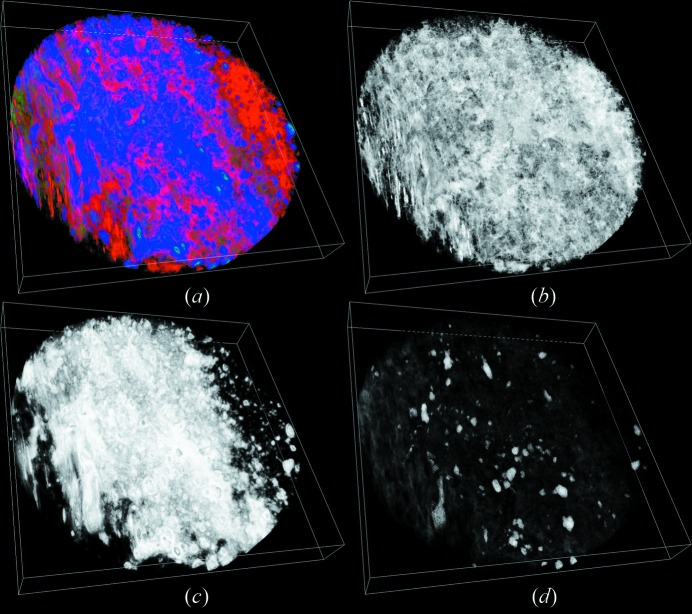
Physical structure of the ROI. In image (*a*), void is displayed as red, minerals of group C are displayed as blue, minerals of group D are displayed as green. Images (*b*), (*c*) and (*d*) are the distributions of void, group C and group D in the ROI, respectively. On a pixel, the displayed intensity of each colour is proportional to the appropriate compositional volume fraction. Co-existence of multiple compositions in a voxel is shown as colour mixing. The size of the ROI is 1489.6 µm × 1489.6 µm × 181.3 µm.

Fig. 8 ▶ shows the physical structure of the ROI in the coal sample. It indicates that the distribution of group C composition tends to take a clustered form, while group D takes a dispersed form and regions of this composition are not connected with each other. The voids are dispersed but connected with each other in the coal sample. It should be pointed out that, although there are a significant numbers of voxels with non-zero void volume fractions, the proportion of void-dominated voxels is small. The void connectivity as shown in the figure is predominately due to such partial void voxels.

The global-CT-based DCM had also been performed to facilitate comparison. In order to minimize the impact of ring artifacts in the global CT (Fig. 3 ▶) and for fine structure display clarification, a sub-region (100 × 100 × 49 voxels) of the ROI with minimal artifacts in global CT was selected for DCM analysis. Consequently, the same sub-region (500 × 500 × 245 voxels) in local CT had been selected for comparison. The DCM computed volume fractions for groups A, B, C and D on the selected sub-region are listed in Table 4 ▶.

**Figure 9:**
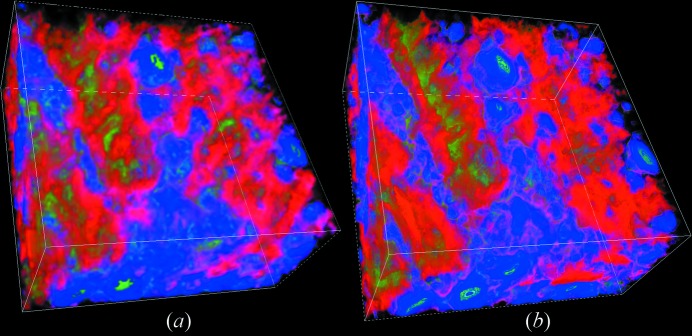
Physical structure of a sub-region in the ROI. Images (*a*) and (*b*) are DCM results based on global and local CT, respectively. In the image, void is displayed as red, minerals of group C are displayed as blue, and minerals of group D are displayed as green. On a pixel, the displayed intensity of each colour is proportional to the appropriate compositional volume fraction. The size of the sub-volume is 370 µm × 370 µm × 181.3 µm.

**Figure 10:**
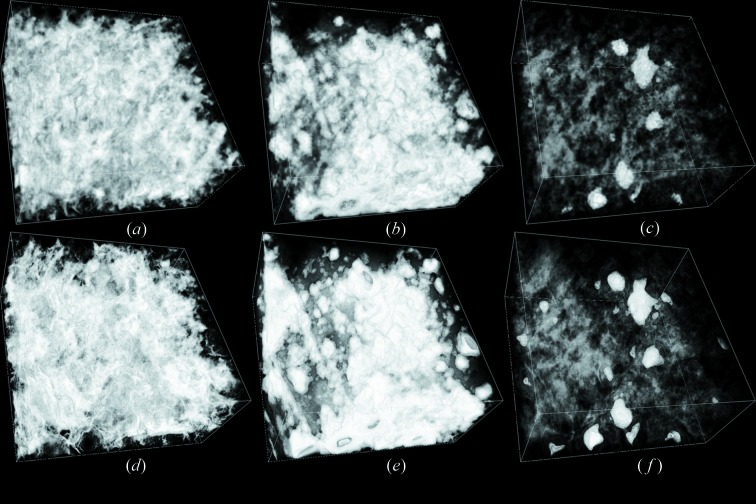
Distributions of void, group C and group D in the sub-region of the ROI. Upper images are global-CT-based DCM results, and lower images are local-CT-based DCM results. (*a*), (*d*) are the distribution of void; (*b*), (*e*) are the distribution of group C; and (*c*), (*f*) are the distribution of group D. The volume fraction is represented by the pixel intensity on the image.

Fig. 9 ▶ shows a three-dimensional view of distributions of void and minerals groups C and D in the ROI sub-region. Fig. 10 shows separate three-dimensional views of void, group C and group D.

The DCM incorporates multiple sets of X-ray CT data as constraints to generate material volume fractions at X-ray CT resolution. The low CT resolution would make it difficult to resolve fine structures of the sample. It causes these fine structures to become blurred or invisible in the DCM results, as can be seen in Figs. 9 ▶ and 10 ▶. Some fine structures are clearly visible in local-CT-based DCM, but they appear to be blurred or have disappeared in global-CT-based DCM. This demonstrates that, although DCM can incorporate effects of fine structures smaller than the CT resolution, its display resolution is restricted by the CT resolution. Higher spatial resolution CT can provide DCM with more spatially resolved constraints, which enables it to display finer structures and give more accurate three-dimensional distributions of compositions in the sample.

The limited resolution also leads to a broadened distribution of the CT-reconstructed values (Yang *et al.*, 2013 ▶). Such a distribution would lead to a small numerical overestimation of void in the coal sample, which is indicated in Table 4 ▶. Table 4 ▶ shows that the average void volume fraction in global CT is clearly bigger than the average void volume fractions in local CT.

## 5. Conclusions   

A quantitative local-CT-based DCM has been developed. The method enables quantitative characterization of three-dimensional distributions of material compositions and void in a ROI of a sample. It has been demonstrated using a coal sample collected from Yangquan mine. The optimal experimental parameters for CT scanning of the coal sample were determined through prior simulations on a numerical phantom and used as a basis for the experimental local-CT measurements. The microscopic physical structure of the ROI in the sample has been determined from this local-CT data using DCM analysis. The material compositions of the sample were categorized into four groups in accordance with their X-ray absorption characteristics. The numerical results of DCM analysis of the local-CT data have been compared with low-resolution global-CT-based DCM.

The results demonstrated the following:

(i) The optimal sample-to-detector distance has been established as 40 mm, and X-ray energies as 18 keV and 24 keV by considering the X-ray energy range and stability on the beamline, experimental convenience and experimental noise level (1%).

(ii) The three-dimensional distributions of four compositions groups including coal matrix, minerals and void in the ROI of the coal sample can be obtained non-destructively with local-CT-based DCM.

(iii) The accuracy of the DCM result is related to CT resolution. Higher-resolution CT enables DCM to produce a more accurate result. Local CT overcomes the limitation of the FOV of the detector, which enables high-resolution CT imaging and accurate DCM modelling.

(iv) Some compositions at length scales smaller than the X-ray CT resolution have been detected and displayed. There were obvious mixings of compositions at the voxel level.

(v) There were a significant amount of voids which were smaller than the X-ray CT resolution. Dispersed sub-voxel-sized voids formed connected clouds. The distribution of group C components takes a clustered form, while group D takes a dispersed form with group D regions not connected to each other.

(vi) Elevated spatial resolution can reduce the over-estimation of void caused by broadened distribution of the CT reconstructed values which is common in CT.

Compared with global-CT-based DCM, the local-CT-based DCM approach has an advantage in resolution. It can display fine structures of compositions in the sample ROI, which is particularly useful when there is a need to see the fine structures in a small region of a large sample.

## Figures and Tables

**Table 1:** List of minerals and their overall volume percentages in the coal sample (reproduced from Wang *et al.*, 2013 ▶)

Mineral name	Molecular formula	Volume percentage
Kaolinite	Al_2_O_3_SiO_2_H_2_O	4.32
Illite	KAl_2_(AlSi_3_O_10_)(OH)_2_	0.23
Quartz	SiO_2_	1.21
Pyrite	FeS_2_	2.60 × 10^−3^
Titania	TiO_2_	0.33
Chlorite	Mg(FeAl)_5_(SiAl)_4_O_10_(OH)_8_	0.077
Dolomite	CaCO_3_MgCO_3_	0.0664
Plagioclase	(NaCa)Al(Al_3_Si)Si_3_	0.0185
Manganese dioxide	MnO_2_	9.26 × 10^−5^
Coal matrix	C_101_H_41_O_4_NS	93.75 (including void volume)

**Table 2:** X-ray total linear absorption and phase-shift coefficients (μ and ϕ) of the main coal compositions (except void) at energies of 12 keV, 18 keV, 24 keV and 30 keV

			12 keV		18 keV		24 keV		30 keV	
Composition	Formula	Density (g cm^−3^)	ϕ (cm^−1^)	μ (cm^−1^)	ϕ (cm^−1^)	μ (cm^−1^)	ϕ (cm^−1^)	μ (cm^−1^)	ϕ (cm^−1^)	μ (cm^−1^)
Kaolinite	Al_2_O_3_SiO_2_H_2_O	2.60	−2293	24.57	−1524	7.565	−1141	3.418	−912.2	1.969
Illite	KAl_2_(AlSi_3_O_10_)(OH)_2_	2.75	−2414	36.79	−1604	11.38	−1201	5.081	−959.9	2.835
Quartz	SiO_2_	2.65	−2336	28.73	−1552	8.824	−1162	3.955	−928.9	2.250
Titania	TiO_2_	4.23	−3567	177.2	−2370	55.81	−1773	24.45	−1416	13.01
Coal matrix	C_101_H_41_O_4_NS	1.36	−1229	2.808	−818.4	1.034	−613.6	0.5862	−490.8	0.4180

**Table 3:** NCCs for different compositions and energies at experimental noise level (1%) when SDD = 40 mm

	Normalized cross-correlation				
Energy (keV)	Kaolinite	Illite	Quartz	Titania	Void
12	0.9927	0.9928	0.9928	0.9907	0.9927
18	0.9926	0.9927	0.9927	0.9929	0.9926
24	0.9924	0.9926	0.9925	0.9930	0.9922
30	0.9921	0.9924	0.9922	0.9929	0.9916

**Table 4:** DCM computed volume fractions

	Overall volume fractions (%)		Overall volume fractions on the sub-region (%)	
Group name	Fig. 7 ▶	Fig. 8 ▶	Local tomography	Global tomography
Group A	3.37	3.69	3.46	4.47
Group B	91.60	91.18	90.70	89.90
Group C	4.93	5.05	5.74	5.59
Group D	0.10	0.08	0.10	0.08

## References

[bb1] Bruening, F. A. & Cohen, A. D. (2005). *Int. J. Coal Geol.* **63**, 195–204.

[bb2] Chen, W., Wang, Y., Liu, H., Deng, B., Yang, Y. & Xiao, T. (2014). *Chin. Opt. Lett.* **12**, 023401.

[bb3] Creelman, R. A. & Ward, C. R. (1996). *Int. J. Coal Geol.* **30**, 249–269.

[bb4] Delaney, A. H. & Bresler, Y. (1995). *IEEE Trans. Image. Process.* **4**, 799–813. 10.1109/83.388081 18290029

[bb5] Faridani, A., Ritman, E. L. & Smith, K. T. (1992). *SIAM J. Appl. Math.* **52**, 459–484.

[bb6] Galbreath, K., Zygarlicke, C., Casuccio, G., Moore, T., Gottlieb, P., Agron-Olshina, N., Huffman, G., Shah, A., Yang, N., Vleeskens, J. & Hamburg, G. (1996). *Fuel*, **75**, 424–430.

[bb7] Gureyev, T., Nesterets, Y., Thompson, D., Wilkins, S., Stevenson, A., Sakellariou, A. & Taylor, J. (2011). *Proc. SPIE*, **8141**, 81410B.

[bb8] Karacan, C. O. & Okandan, E. (2001). *Fuel*, **80**, 509–520.

[bb9] Katsevich, A. I. & Ramm, A. G. (1996). *SIAM J. Appl. Math.* **56**, 167–191.

[bb10] Ketcham, R. A. & Carlson, W. D. (2001). *Comput. Geosci.* **27**, 381–400.

[bb11] Kudo, H., Courdurier, M., Noo, F. & Defrise, M. (2008). *Phys. Med. Biol.* **53**, 2207–2231. 10.1088/0031-9155/53/9/001 18401067

[bb12] Kyrieleis, A., Titarenko, V., Ibison, M., Connolley, T. & Withers, P. J. (2011). *J. Microsc.* **241**, 69–82. 10.1111/j.1365-2818.2010.03408.x 21118206

[bb13] Lawrie, G. A., Gentle, I. R., Fong, C. & Glikson, M. (1997). *Fuel*, **76**, 1519–1526.

[bb14] Levine, D. G., Schlosberg, R. H. & Silbernagel, B. G. (1982). *Proc. Natl Acad. Sci. USA*, **79**, 3365–3370.

[bb15] Lewitt, R. M. (1979). *Med. Phys.* **6**, 412–417. 10.1118/1.594519 492075

[bb16] Lewitt, R. M. & Bates, R. H. T. (1978). *Optik*, **50**, 189–204.

[bb17] Mayo, S. C., Tulloh, A. M., Trinchi, A. & Yang, S. Y. (2012). *Microsc. Microanal.* **18**, 524–530. 10.1017/S1431927612000323 22640963

[bb18] Natterer, F. (1986). *The Mathematics of Computerized Tomography*, 1st ed. New York: Wiley.

[bb19] Paganin, D., Mayo, S. C., Gureyev, T. E., Miller, P. R. & Wilkins, S. W. (2002). *J. Microsc.* **206**, 33–40. 10.1046/j.1365-2818.2002.01010.x 12000561

[bb20] Saikia, B. K. & Ninomiya, Y. (2011). *Fuel Process. Technol.* **92**, 1068–1077.

[bb21] Trinchi, A., Yang, Y. S., Huang, J. Z., Falcaro, P., Buso, D. & Cao, L. Q. (2012). *Modell. Simul. Mater. Sci. Eng.* **20**, 015013.

[bb22] Vassilev, S. V. & Tascón, J. M. D. (2003). *Energ. Fuel.* **17**, 271–281.

[bb23] Wang, H. P., Yang, Y. S., Wang, Y. D., Yang, J. L., Jia, J. & Nie, Y. H. (2013). *Fuel*, **106**, 219–225.

[bb24] Xiao, T., Bergamaschi, A., Dreossi, D., Longo, R., Olivo, A., Pani, S., Rigon, L., Rokvic, T., Venanzi, C. & Castelli, E. (2005). *Nucl. Instrum. Methods Phys. Res. A*, **548**, 155–162.

[bb26] Yang, S., Furman, S. & Tulloh, A. (2008). *Adv. Mater. Res.* **32**, 267–270.

[bb27] Yang, S., Gao, D., Muster, T., Tulloh, A., Furman, S., Mayo, S. & Trinchi, A. (2010*b*). *Mater. Sci. Forum*, **654**–**656**, 1686–1689.

[bb25] Yang, Y. S. (2012). *Information Engineering Research Institute*, *Lecture Notes on Information Technology*, Vol. 15, pp. 198–205.

[bb28] Yang, Y. S., Gureyev, T. E., Tulloh, A., Clennell, M. B. & Pervukhina, M. (2010*a*). *Meas. Sci. Technol.* **21**, 1–6.

[bb29] Yang, Y. S., Liu, K. Y., Mayo, S., Tulloh, A., Clennell, M. B. & Xiao, T. Q. (2013). *J. Petrol. Sci. Eng.* **105**, 76–83.

[bb30] Yang, Y. S., Tulloh, A., Cole, I., Furman, S. & Hughes, A. (2007). *J. Aust. Ceram. Soc.* **43**, 159–164.

[bb31] Yang, Y. S., Wang, H. P. & Gao, J. R. (2012). *J. Shanxi Univ.* **35**, 248–254.

[bb32] Yao, Y. B., Liu, D. M., Che, Y., Tang, D. Z., Tang, S. S. & Huang, W. H. (2009). *Int. J. Coal Geol.* **80**, 113–123.

